# Experimental Pain and Opioid Analgesia in Volunteers at High Risk for Obstructive Sleep Apnea

**DOI:** 10.1371/journal.pone.0054807

**Published:** 2013-01-29

**Authors:** Anthony G. Doufas, Lu Tian, Kevin A. Padrez, Puntarica Suwanprathes, James A. Cardell, Holden T. Maecker, Periklis Panousis

**Affiliations:** 1 Department of Anesthesiology, Perioperative and Pain Medicine, Stanford University School of Medicine, Stanford, California, United States of America; 2 Health Research and Policy, Stanford University School of Medicine, Stanford, California, United States of America; 3 School of Medicine, University of California San Francisco, San Francisco, California, United States of America; 4 Stanford Sleep Medicine Center, Stanford University School of Medicine, Stanford, California, United States of America; 5 Department of Microbiology and Immunology, and Human Immune Monitoring Center, Stanford University School of Medicine, Stanford, California, United States of America; 6 Outcomes Research Consortium, the Cleveland Clinic, Cleveland, Ohio, United States of America; Charité University Medicine Berlin, Germany

## Abstract

**Background:**

Obstructive sleep apnea (OSA) is characterized by recurrent nocturnal hypoxia and sleep disruption. Sleep fragmentation caused hyperalgesia in volunteers, while nocturnal hypoxemia enhanced morphine analgesic potency in children with OSA. This evidence directly relates to surgical OSA patients who are at risk for airway compromise due to postoperative use of opioids. Using accepted experimental pain models, we characterized pain processing and opioid analgesia in male volunteers recruited based on their risk for OSA.

**Methods:**

After approval from the Intitutional Review Board and informed consent, we assessed heat and cold pain thresholds and tolerances in volunteers after overnight polysomnography (PSG). Three pro-inflammatory and 3 hypoxia markers were determined in the serum. Pain tests were performed at baseline, placebo, and two effect site concentrations of remifentanil (1 and 2 µg/ml), an μ-opioid agonist. Linear mixed effects regression models were employed to evaluate the association of 3 PSG descriptors [wake after sleep onset, number of sleep stage shifts, and lowest oxyhemoglobin saturation (SaO_2_) during sleep] and all serum markers with pain thresholds and tolerances at baseline, as well as their changes under remifentanil.

**Results:**

Forty-three volunteers (12 normal and 31 with a PSG-based diagnosis of OSA) were included in the analysis. The lower nadir SaO_2_ and higher insulin growth factor binding protein-1 (IGFBP-1) were associated with higher analgesic sensitivity to remifentanil (SaO_2_, P = 0.0440; IGFBP-1, P = 0.0013). Other pro-inflammatory mediators like interleukin-1β and tumor necrosis factor-α (TNF-α) were associated with an enhanced sensitivity to the opioid analgesic effect (IL-1β, P = 0.0218; TNF-α, P = 0.0276).

**Conclusions:**

Nocturnal hypoxemia in subjects at high risk for OSA was associated with an increased potency of opioid analgesia. A serum hypoxia marker (IGFBP-1) was associated with hypoalgesia and increased potency to opioid analgesia; other pro-inflammatory mediators also predicted an enhanced opioid potency.

**Trial Registration: ClinicalTrials.gov:**

NCT00672737.

## Introduction

Obstructive sleep apnea (OSA) is a common health condition [1], [2] characterized by cyclic cessations of airflow due to intermittent complete or partial airway obstruction, leading to episodic hypoxia and repeated arousals from sleep [3]. It is estimated that approximately 20% of the general [2] and surgical [4] population suffer from OSA with a fraction up to 80% of these individuals lacking a formal diagnosis [4], [5]. Sleep-disordered breathing has been linked to cardiovascular [6], [7] and metabolic [8] morbidity, while accumulating evidence suggests that OSA might increase the risk for respiratory complications [9]–[14] in the postoperative period due to drug-induced airway compromise, especially when μ-opioid receptor agonists are administered to treat pain [15]–[17]. It is thus important to delineate the relationship between sleep-disordered breathing, pain behavior, and opioid analgesia because this would facilitate opioid titration and thus safer postoperative pain management in these patients.

Important phenotypic components of sleep-disordered breathing, like sleep disruption, recurrent nocturnal hypoxemia, and systemic inflammation [18], have been linked to altered pain processing. Both experimental [19]–[21] and clinical [22], [23] evidence suggest that inadequate and/or disrupted sleep could enhance pain sensitivity in humans, while prolonged deprivation of sleep promoted the expression [24] and release of major sleep-regulating cytokines, including tumor necrosis factor (TNF)-α, interleukin (IL)-1β, and IL-6 [25]–[30], which are known to exert hyperalgesic effects in various experimental models [31], [32]. Consistent with these observations, treatment of OSA with continuous positive airway pressure (CPAP), presumably enhancing sleep continuity, decreased the sensitivity to painful stimuli in adults [33]. On the other hand, children suffering from OSA demonstrated an increased sensitivity to the postoperative analgesic effect of morphine; interestingly, morphine dose requirement for postoperative pain was inversely proportional to the degree of nocturnal hypoxemia preoperatively [34], [35].

Herein, we evaluated experimental pain processing and opioid analgesia in male volunteers suffering from sleep-disordered breathing. We examined the effect of sleep disruption and nocturnal hypoxemia on pain behavior and opioid analgesia. We hypothesized that nocturnal hypoxemia would be associated with a decreased sensitivity to painful stimuli and increased potency to opioid analgesia, whereas sleep disruption would enhance sensitivity to pain. In this context we also investigated the role of specific inflammatory and hypoxia markers in predicting sensitivity to pain and opioid analgesic effect.

## Methods

### Ethics Statement

The Stanford Research Compliance Office (Human Subjects Research and IRB: humansubjects.stanford.edu) approved the study. All participants gave written informed consent, while all procedures were conducted according to the principles expressed in the Declaration of Helsinki.

### Subjects Selection

Epidemiologic evidence shows that male gender [1] and habitual snoring [1], [36] are among the known risk factors for the presence of obstructive sleep apnea (OSA) in the general population. Thus, from January 2008 till March 2010, we invited male volunteers (18–55 years old) with a history of habitual snoring and/or a formal diagnosis of OSA to participate in a study evaluating sleep and experimental pain processing.

We excluded volunteers with a body mass index (BMI) equal or higher than 35 Kg/m^2^, history of diabetes, pulmonary or heart conditions that could affect oxygenation status, chronic pain condition, psychiatric disease, moderate to severe depression (based on Beck depression inventory), untreated thyroid disease, and those who were taking analgesics or other drugs affecting the central nervous system, including antidepressants. Also, we excluded volunteers who had been treated for their OSA with continuous positive airway pressure (CPAP). The trial was registered at the ClinicalTrials.gov under the title “study of pain processing in subjects suffering from obstructive sleep apnea” (NCT00672737, PI: AG Doufas).

### Protocol

The study was conducted in two separate days, approximately one week apart. In the first day, volunteers underwent overnight polysomnography (PSG) at home or in a special research facility of the Sleep Clinic at Stanford Center for Human Sleep Research. Approximately one week later, they were subjected to quantitative sensory testing at the Human Pain Laboratory in the Department of Anesthesiology, Perioperative and Pain Medicine at Stanford. The volunteers were not aware of the study hypothesis and they were informed the results of the PSG assessment only after the end of the trial.

On the second study day, the volunteers fasted for at least 6 hours and arrived at the laboratory at 8:30 AM. After undergoing a basic physical examination and completing a series of questionnaires (see Measurements), a 20-g catheter was inserted at the antecubital fossa of the left arm and a 10-mL blood sample was obtained to measure serum glucose, glycated hemoglobin (HbA1c), 3 pro-inflammatory cytokines, and 3 hypoxia markers.

Standard cardio-respiratory monitors including oscillometric blood pressure, electrocardiogram, and pulse oximetry (SaO_2_) were applied to the volunteers. Normothermia was maintained with forced-air warming, while volunteers breathed supplemental oxygen via nasal prongs to maintain a SaO_2_ greater than 90%.

After a short training period, quantitative sensory testing for heat and cold was performed at four different phases: baseline, placebo, and at two different effect site concentrations (Ce) of remifentanil. Placebo was always administered first, while the order of remifentanil Ce was randomized between 2 and 4 µg/ml in the first 9 volunteers and between 1 and 2 µg/ml in the rest. The reason for this change in the testing remifentanil concentrations was that in several subjects the predefined maximum values for heat (52°C) and cold (180 sec) painful stimuli (see below) were reached when a Ce = 4 µg/ml was targeted.

The drug delivery system consisted of a Harvard 22 (Harvard Apparatus, Holliston, MA) electronic syringe pump and the STANPUMP software driver (freely available by Steve L. Shafer, MD), based on a previously developed [37] and validated [38] pharmacokinetic-pharmacodynamic model for remifentanil. Volunteers were blinded to the pump function, during all phases of the experiment, while the investigator performing the sensory testing and sedation assessment was blinded to the remifentanil Ce and PSG recordings. During the placebo and the two remifentanil Ce phases of the experiment volunteers were told that they may or may not be administered an active drug.

Quantitative sensory testing for heat and cold with a 10-min-long resting interval in-between, begun approximately 10 minutes after the initiation of the simulated or actual remifentanil in order to make sure that a pseudo-steady state has been achieved. At the end of each of the four phases vital signs and sedation, using the responsiveness component of the observer’s assessment alertness/sedation (OAA/S) scale [39], were recorded.

At the end of the experiment the remifentanil infusion was stopped and volunteers left to recover completely before they were discharged home.

**Figure 1:**
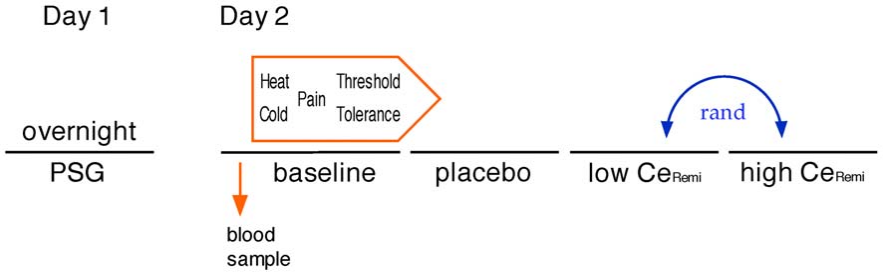
Study flow scheme. On the first study day, the volunteers underwent home-based or in-hospital overnight polysomnography (PSG). Approximately a week later, they underwent experimental pain testing after a blood sample was withdrawn for determination of pro-inflammatory and hypoxia markers. Threshold and tolerances were determined for both heat and cold painful stimuli at baseline (no drug), placebo, and two (low and high) effect site concentrations (Ce) of remifentanil (1 and 2 µg/ml, or 2 and 4 µg/ml) that were targeted using an electronic syringe pump connected to a laptop equiped with a special software driver (STANPUMP, written by Steve L. Shafer, MD) and a pharmacokinetic-pharmacodynamic model for remifentanil. The order of presentation for the two remifentanil Ce was randomized, using special software and sealed envelopes that were opened on the day of trial.

A concise study flow diagram is presented in Figure 1.

#### Protocol deviations

We recruited volunteers with a BMI greater than the originally set upper limit of 29 kg/m^2^, due to difficulty in recruiting overweight (BMI<30 kg/m^2^) subjects that would also demonstrate the phenotypic characteristics or have a formal diagnosis of obstructive sleep apnea. Consequently, we have recruited 9 volunteers with a BMI>29 kg/m^2^; all were lower than or equal to 32.66 kg/m^2^.

Several originally planned assessments and/or procedures were not included in the analysis or reported results, either because of insufficient data collection or because the evaluated outcomes were irrelevant to the aim of the present project. These assessments include heart rate variability, instantaneous sleepiness using the Karolinska sleepiness scale (KSS), and assessment of vigilance using the psychomotor vigilance test (PVT).

### Measurements

The volunteers’ demographic and morphometric characteristics were recorded. Heart rate from the electrocardiogram (ECG), blood pressure, respiratory rate via ECG and direct observation, and arterial oxyhemoglobin saturation (pulse oximetry, SaO_2_) were continuously monitored and recorded at the end of each of the four phases of the experiment, along with the responsiveness component of the OAA/S score.

#### Polysomnography

Volunteers underwent an overnight polysomnography either at their home or in a sleep research facility, depending on the availability of the latter. In the first case, a type II [40] portable sleep recording device (Crystal Monitor 20-B, CleveMed, Cleveland Medical Devices, Inc., Cleveland, OH) was used to perform unattended home-based polysomnography (PSG). To ensure that maximal sleep was attained, approximately 2 hours prior to their usual bedtime, the volunteers arrived at the study center where they were connected to the PSG monitor by a certified polysomnography technologist. Physiological signals for a comprehensive PSG assessment were acquired through 14 different channels, including electroencephalogram (EEG; A1, A2 C3, O2), electrocardiogram (ECG), chin and leg electromyogram (EMG), electro-oculogram (EOG), nasal air flow, snore, thoracic and abdominal respiratory effort, and pulse oximetry (SaO_2_). Subjects returned to their home with the device firmly connected and were instructed to turn on the device as they lay down for bed and turn off the device after they were out of bed in the morning. The data was recorded on a removable SD memory card. Subjects returned with the device the following morning where it was safely removed by the technician.

Alternatively, volunteers arrived at the sleep research facility approximately 2.5 hours prior to their usual bedtime in order to allow time for adequate preparation. A certified polysomnography technologist connected the volunteers to a laboratory-based PSG equipment using the same channels and as the home-based PSG device. Signals were monitored and acquired using Alice software (Alice Sleepware 2.8.78, Philips Respironics, Murrysville, PA). More specifically, a standard diagnostic PSG study was conducted according to practice guidelines published by the American Academy of Sleep Medicine (AASM) [41], with recording electrodes and equipment attached to the volunteer to enable monitoring of the EEG (C3-A2 and C4-A1, O2-A1 and O1-A2), EOG (ROC-A1, LOC-A2), chin and anterior tibialis EMG, heart rate by two-lead ECG, snoring intensity (anterior neck microphone), nasal pressure (nasal cannula), oral airflow (thermocouple), thoracic and abdominal movement (inductance plethysmography bands), and SaO_2_.

Polysomnography recordings were scored in 30-sec epochs. Sleep staging was based on the Rechtschaffen and Kales criteria [42], while EEG arousals were scored according to published rules [43]. Apnea and hypopnea events were determined using the criteria for clinical research proposed by the AASM [44]. More specifically, apnea was defined by a more than 90% decrease from baseline in the amplitude of the nasal pressure signal lasting ≥10 sec. Hypopneas were determined by a clear reduction (>50% but ≤90%) in the amplitude of the signal from its baseline value, or if the decrease in the amplitude of the signal was <50%, but it was associated with either an oxygen desaturation >3% or an arousal, and the event duration was ≥10 seconds.

Polysomnographic descriptors of sleep included the total sleep period (TSP, time in bed with lights off), total sleep time (TST), sleep efficiency expressed in % [total time in bed sleeping, SE = (TST/TSP) * 100], sleep onset latency (SOL; time between lights off and falling asleep), sleep stages 1, 2, and 3 plus 4 (combined; all as percentages of TST), rapid eye movement (REM) sleep stage, number of REM periods, number of sleep stage shifts, wake after sleep onset (WASO; time spent awake after falling asleep) number of awakenings, apnea/hypopnea index (AHI; the sum of all apneic and hypopneic events divided by the hours of TST), nadir arterial oxyhemoglobin saturation (SaO_2_, pulse oximetry), and time spent with an SaO_2_<90% (in % of TST). PSG studies with sleep efficiency lower than 50% were excluded from the analysis.

All PSG studies were scored manually using Sandman software (Sandman Elite 8.0, Covidien, Boulder, CO) according to the criteria for clinical research published by the AASM [44], by a sleep physiologist who was blinded to the volunteers’ medical history. Polysomnography always preceded quantitative sensory testing by approximately one week. When volunteers fulfilled the criteria for diagnosis of sleep-disordered breathing (SDB), they were informed accordingly at the end of the study, so that they could follow up on their condition.

#### Scales and questionnaires

The presence of signs and symptoms that are compatible with the diagnosis of obstructive sleep apnea (OSA) [45], [46], including snoring, unintentional sleep during wakefulness, daytime sleepiness, non-restorative sleep, fatigue, waking up breath holding, gasping, or choking, and/or having witnessed apneas, was evaluated using the questionnaire (10 yes/no questions) for exploring obstructive sleep apnea hypopnea syndrome (OSAHS) symptoms, proposed by the American Academy of Sleep Medicine [47]. Although clinical screening can increase confidence in the diagnosis of OSA (especially when 5≤ AHI<15), the absence of these findings cannot be used to exclude such a diagnosis.

Habitual sleepiness was assessed using the Epworth sleepiness scale (ESS) [48], which measures the general level of daytime sleepiness by rating the tendency of subjects to doze off or fall asleep (0 for “no chance of dozing” to 3 for “high chance of dozing”) when in 8 different situations commonly encountered in daily life.

Mood was assessed using the Beck depression inventory (BDI) [49]. According to BDI, 21 different statements were scored between 0 (indicating no depression) and 3 (indicating severe depression), with a total score up to 20 indicating none to mild depression.

Sedation level was evaluated at the end of each of the four experimental phases, using the responsiveness component of the observer’s assessment alertness/sedation (OAA/S) scale: (5) when “Responds readily to name spoken in normal tone”, (4) for “lethargic response to name spoken in normal tone”, (3) when “responds only after name is spoken loudly and/or repeatedly”, (2) when “responds only after mild prodding or shaking”, and (1) when “does not respond to mild prodding or shaking” [39].

#### Inflammation and hypoxia markers

On the second study day and before the initiation of quantitative sensory testing, we determined in the volunteers’ serum the concentration of 9 pro-inflammatory (granulocyte macrophage colony-stimulating factor, GM-CSF; interferon-gamma, IFN-γ; interleukin-10, IL-10; IL-12p70; IL-1β; IL-2; IL-6; IL-8; tumor necrosis factor-α, TNF-α) and 3 hypoxia (erythropoietin, EPO; insulin growth factor binding protein-1, IGFBP-1; vascular endothelial growth factor, VEGF) markers.

Assays kits were purchased from Meso Scale Discovery (Gaithersburg, MD) and the protocol was followed as recommended. Briefly, antibody-coated 96-well plates were pre-incubated for 30 minutes with 25 ml/well of Diluent 2. Sample or 7-point diluted Calibrators were added and plates were sealed and incubated at room temperature for 2.5 h with shaking. Plates were aspirated and washed three times with PBST (PBS +0.05% Tween-20), while 25 ml of Sulfo-Tag secondary antibody in Diluent 3 were then added and the plates incubated for 2.5 h with shaking. Plates were washed 3 times in PBST as above and 150 ml of 2X Reading Buffer were added. Plates were read immediately in a Sector Imager 2400 (MesoScale Discovery). Results were analyzed with Masterplex software (MiraiBio Group, Hitashi Corporation).

For the 3-plex hypoxia kit, the following changes apply: Plates were pre-blocked with 150 ml of Blocker C for 1 h at room temperature with shaking, followed by PBST washes as above. Twenty-five milliliters per well of Diluent 7 were added followed by either sample or Calibrators. The Sulfo-Tag antibody was diluted in Diluent 8 and the assay was completed as outlined above except that 1X Reading Buffer was used.

Standards were fitted to 5-parameter logistic regression curves, from which concentrations of test samples were estimated. R^2^ values for the fitted curves were >0.98 for all markers. Lower limits of detection were as follows (in pg/mL): GM-CSF, 0.16; IFN-γ, 0.28; IL-10, 0.09; IL-12p70, 0.65; IL-1β, 0.01; IL-2, 0.50; IL-6, 0.14; IL-8, 0.01; TNF-α, 0.54; IGFBP-1, 20.32; EPO, 2.19; VEGF, 12.99. Spike-recovery experiments (not shown) using the S6 Calibrator yielded recovery of 79–112% for all cytokines diluted in serum versus standard buffer.

#### Quantitative sensory testing

Experimental sensory testing for heat and old was performed in a 25-°C silent room, appropriate for psychophysical assessment, with the volunteers comfortably seated on a reclining chair. Both Aδ and C nociceptive fibers are responsible for encoding and relaying centrally the noxious cold and noxious heat stimuli [50]. The respective nociceptors demonstrate response characteristics that correlate well with psychophysical pain ratings during the application of noxious thermal stimuli [51]–[53]. Heat pain and cold pressor tests were performed in the same order with a 10-min-long resting interval in-between, at each of the four phases of the experiment (baseline, placebo, and two remifentanil concentrations). A short training period consisted of 2–3 assessments for each of the heat and cold pain parameters preceded the actual testing phase of the experiment.

#### Heat pain test

A thermal sensory analyzer (TSA II Neurosensory Analyzer; Medoc Advanced Medical Systems, Durham, NC) was used to administer heat stimuli via a 30×30 mm thermode in contact with skin of the volar left forearm. According to the method of limits [54], a pressurized air-driven vortex stimulator raised the temperature of the thermode at a rate of 1°C/sec from a baseline of 35°C until the patient pushed the button of a hand-held device (computer “mouse”) to indicate that the perception changed from very hot to painful. This procedure was repeated at least 3 times and until the difference among individual threshold values was less than 0.5°C. All the obtained temperature values were recorded for the analysis. A similar procedure was used to determine the thermode temperature causing maximum tolerable pain (tolerance). After each individual testing session the location of the thermode was slightly changed to avoid sensitization of the skin to heat. The inter-stimulus interval was 30 seconds. The maximum thermode temperature was limited to 52°C to prevent tissue damage.

#### Cold pressor test

A standard commonly used procedure was followed to determine pain sensitivity to cold stimuli [55]. Subjects were asked to immerse one hand and forearm into a container filled with 12 L of 0–1-°C ice water. To maintain testing consistency among subjects, care was taken to assure that the whole palm stayed in full contact with the bottom of the container throughout the immersion. A pump steadily circulated the ice water, while the temperature was continuously monitored using an electronic thermometer. Volunteers were asked to verbally indicate when they first detected pain and remove their hand when the pain could no longer be tolerated. The time elapsed between hand immersion to pain detection (threshold) and hand withdrawal (tolerance) was recorded. Cold pain threshold and tolerance were measured at least twice, or till two measurements were within a 20-%-range. The inter-stimulus interval was 5 min. The maximum time the hand was allowed immersed in ice water was 180 sec to avoid any cold-related skin injury.

### Data Analysis

All data were presented as medians (ranges), or number (%) of volunteers. Age, body mass index (BMI), fasting morning glucose, glycated hemoglobin (HbA1c), Epworth sleepiness scale (ESS) and Beck depression inventory (BDI) scores, sleep descriptors (PSG), and cytokine serum levels were compared between subjects with an apnea/hypopnea index (AHI) ≥5 events per hour of sleep and those with AHI<5, two-sample Mann-Whitney rank tests. The chi-square test, whose null distribution was generated via permutation, was used to compare the distribution of race between the two groups.

#### Mixed effects linear regression model

For each of the four pain-related variables (heat pain threshold, heat pain tolerance, cold pain threshold, and cold pain tolerance), we fit a linear mixed-effects regression model:
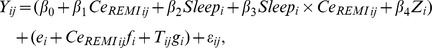
where *Y_ij_* is the response for the *i_th_* patient in the *j_th_* experiment, *Ce_REMI ij_* is the targeted remifentanil effect site concentration level [i.e., 0 (baseline and placebo), 1, 2, and 4 µg/ml remifentanil Ce) used in the *i_th_* patient in *j_th_* experiment, *Sleep_i_* represents PSG descriptors used in the analysis (i.e., wake after sleep onset time, number of stage shifts, and nadir SaO_2_) for the *i_th_* patient, *T_i_* is a binary covariate indicating if the *i_th_* patient received intervention (i.e., any drug treatment including placebo) in the *j_th_* experiment and *Z_i_* represents confounding factors adjusted in the regression analysis (see below). The subject-specific random intercept, remifentanil effect, and placebo effect (*e_i_*, *f_i_*, *g_i_*), were assumed normally distributed and used to account for the potential correlations between repeated pain-related measurements for the same volunteer. In this model, *β_2_* and *β_3_* measure the effect of sleep (PSG) descriptors on the pain response and on the remifentanil effect on pain response (i.e., elevation of pain threshold/tolerance), respectively. In the first set of analyses, we adjusted the model for the BMI, age, and a binary variable indicating the type (home-based vs. in-laboratory) of PSG study (*Z_i_*), as potential confounders, while in the second set of analyses we additionally adjusted for the inflammation and hypoxia markers measured by IL-6, IL-1β, TNF-α, and IGFBP-1, VEGF, EPO, respectively. The purpose of adjusting for systemic inflammation and hypoxia-related markers was to demonstrate if a potential effect of sleep fragmentation or recurrent hypoxemia on pain behavior and/or opioid analgesia was mediated through changes in the inflammatory or hypoxia markers, respectively. We selected the specific sleep and pro-inflammatory descriptors based on prior literature evidence [19], [21], [24], [34], [35], [56]–[58] and the results of non-paired comparisons between OSA and non-OSA subjects, described above. The statistical significance level was set at 0.05.

All statistical analyses were performed using R version 2–10-1 (The R Foundation for Statistical Computing; http://www.R-project.org).

## Results

**Figure 2:**
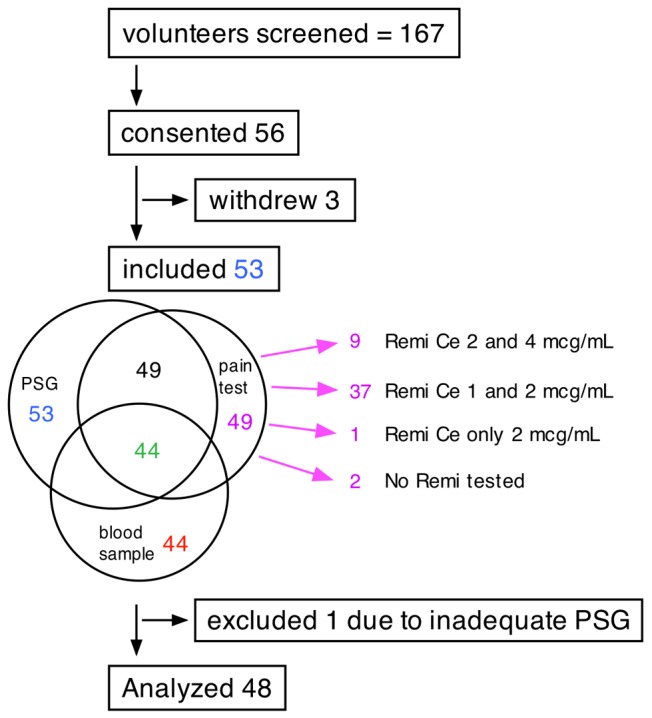
Volunteers and study procedures. A total of 167 volunteers were screened via telephone or a personal interview. Fifty six volunteers signed an informed concent; 3 of them withdrew before participation in any of the study procedures. All remaining 53 underwent an overnight polysomnography, but only 49 came for the pain test on the second study day. Blood was not drawn from 3 volunteers due to technical difficulties, while 2 more blood samples were lost before analysis was done; a total of 44 blood samples provided data on inflammatory and hypoxia markers. The first 9 volunteers were tested on remifentanil Ce of 2 and 4 µg/ml and all the remaining on 1 and 2 µg/ml. Two volunteers experienced intence nausea; one at the end and the other at the beginning of the first remifentanil Ce (2 µg/ml); the experiment was stopped in both cases, but pain-related measurements were acquired only in the first case. A pump malfunction prohibited the administration of remifentanil in 1 subject. One volunteer was excluded from the analysis due to an inadequate sleep study. Finally, data from 48 volunteers were included in the analysis.

Figure 2 presents the recruitment and inclusion scheme of the volunteers to the various study procedures. A total of 49 volunteers underwent both a polysomnography (PSG) study and experimental pain testing while in 44 of them blood was also withdrawn for determining pro-inflammatory and hypoxia markers. After excluding one volunteer who had an inadequate PSG study, we included data from 48 subjects in the final analysis.

Thirteen (7 with an AHI<5 events/h) out of the 48 subjects underwent a home-based, unattended PSG study. With the exception of 2 subjects, all participants (N = 31) with an AHI≥5 responded positively to at least 3 (median No of “yes” = 7) out of the 10 questions exploring OSAHS, thus raising the probability for diagnosis of OSA.

During all the phases of the experiment cardiorespiratory parameters remained essentially unchanged from their baseline levels. Besides an OAA/S score of 3 (responds only after name is spoken loudly and/or repeatedly) that was encountered in only 2 occasions under remifentanil concentration of Ce = 4 µg/ml, volunteers were able to respond either readily or lethargically to their name spoken in normal tone (i.e., an OAA/S score of 5 or 4, respectively). Baseline comparisons between volunteers with (AHI≥5 events/h) and without (AHI<5) a PSG-based diagnosis of OSA revealed no significant differences in the morphometrics and demographics, metabolic, mood, sleepiness, and pro-inflammatory markers (Table 1). Non-paired comparisons of sleep descriptors between OSA and non-OSA volunteers revealed significantly “lighter” and more discontinuous sleep with a greater amount of stage 1 sleep (P = 0.019), higher number of stage shifts (P = 0.006), more WASO time (P = 0.006), and higher number of awakenings (P = 0.005) in the former, compared to the latter. In addition, volunteers with an AHI≥5 events/hour or sleep presented with significantly more arterial oxyhemoglobin desaturation with a lower nadir SaO_2_ (P<0.0001) and a longer period of total sleep time spent at an SaO_2_<90% (P<0.0001), compared to those with AHI<5 (Table 2).

**Table 1:** Baseline morphometrics and demographics, metabolic, sleepiness, mood, and pro-inflammatory markers.

Volunteers	AHI<5(N = 15)	AHI≥5(N = 33)	P value
**Age (yr)**	31 (19–52)	34 (19–54)	0.436
**Body Mass Index (kg/m^2^)**	24 (20–32)	26 (20–33)	0.070
**RACE**			
** White (%)**	8 (53)	20 (61)	0.930
** Hispanic (%)**	1 (7)	4 (12)	
** Asian (%)**	2 (13)	4 (12)	
** African American (%)**	1 (7)	1 (3)	
** Other (%)**	3 (20)	4 (12)	
**Fasting Glucose (mg/dl)**	91 (74–103)	92 (74–123)	0.552
**HbA1c (%)**	5.3 (4.6–6.4)	5.4 (4.7–6.5)	0.679
**Epworth Sleepiness Scale**	8 (3–15)	9 (1–17)	0.892
**Beck Depression Inventory**	3 (0–17)	4 (0–15)	0.929
**INFLAMMATION MARKERS**	**(N = 12)**	**(N = 31)**	
** IL - 6 (pg/ml)**	0.59 (0.26–0.78)	0.69 (0.34–2.59)	0.015
** IL - 1β (pg/ml)**	0.12 (0.01–2.39)	0.26 (0.01–5.31)	0.154
** TNF - α (pg/ml)**	7.77 (4.57–14.57)	7.88 (5.39–31.68)	0.818
**HYPOXIA MARKERS**			
** IGFBP - 1 (pg/ml)**	3190 (663–11710)	2147 (375.5–12996)	0.636
** EPO (pg/ml)**	7.86 (3.71–14.06)	10.43 (4.39–17.79)	0.139
** VEGF (pg/ml)**	241.5 (21.44–805.4)	405.1 (97.95–1409)	0.061

Age, body mass index (BMI), ethnicity, and baseline fasting morning glucose, glycated hemoglobin (HbA1c), Epworth sleepiness scale (ESS), and Beck depression inventory (BDI) for the 48 volunteers. Inflammation and hypoxia markers were determined in 44 out of the 48 volunteers, but only 43 of them were included in the analysis. All parameters are summarized as medians (range) separately for the volunteers with (apnea/hypopnea index, AHI≥5 events/h, N = 33) and without (AHI<5, N = 15) a PSG-based diagnosis of obstructive sleep apnea (OSA). Two volunteers had a BMI>30 kg/m^2^, each belonging to each AHI class. Comparisons between subjects with and without OSA were performed with an appropriate non-paired test, while distribution of race was assessed by X^2^ test; alpha level was set at 0.05.

**Table 2:** Sleep architecture and breathing based on overnight polysomnography.

	AHI<5(N = 15)	AHI≥5(N = 33)	P value
**Total sleep period (min)**	417 (251–531)	438 (233–540)	0.436
**Total sleep time (min)**	388 (232–489)	365 (184–5 15)	0.711
**Sleep efficiency (%)**	88 (55–93)	81 (67–94)	0.171
**Sleep onset latency (min)**	18 (5–179)	14 (0.5–114)	0.571
**Stage 1 (% TST)**	5.2 (2–8.6)	6.9 (1.7–23.7)	0.019
**Stage 2 (% TST)**	73.6 (0.08–82.8)	70.2 (46.3–86.2)	0.896
**Stage 3 and 4 (% TST)**	1.9 (0–14.1)	0.8 (0–18.5)	0.973
**Stage REM (% TST)**	17.2 (8.5–27.2)	16.2 (0–32.7)	0.335
**Number of REM periods**	4 (1–12)	4 (0–12)	0.376
**Number of stage shifts**	89 (34–110)	98 (54–224)	0.006
**WASO (min)**	28.7 (10.6–93)	52.8 (17.5–153)	0.006
**Number of awakenings**	28 (11–34)	31 (15–86)	0.005
**AHI (events/h)**	2.4 (0–4.9)	13 (5–66)	<0.0001
**Nadir SaO_2_ (%)**	93 (87–96)	87 (73–94)	<0.0001
**SaO_2_<90% (% TST)**	0 (0–0.34)	0.12 (0–11.09)	0.003
**Awake baseline SaO_2_**	99 (96–100)	98 (95–100)	0.0642

Seep efficiency represents the percentage of the total sleep period that was spent asleep; different sleep stages are expressed as percentages of the total sleep time (TST); WASO stands for “wake after sleep onset”, and AHI for “apnea/hypopnea index”; all parameters are expressed as medians (range). All parameters are summarized separately for those with (apnea/hypopnea index, AHI≥5 events/h, Ν = 33) and without (AHI<5, Ν = 15) a PSG-based diagnosis of obstructive sleep apnea (OSA). Non-paired comparisons between subjects with and without OSA were performed with an appropriate test; alpha level was set at 0.05.

### Mixed effects linear regression models

In this analysis we included only the 43 volunteers with a complete set of data (i.e., polysomnography, pain testing, and pro-inflammatory markers).

Insulin growth factor binding protein-1 (IGFBP-1) in the serum was a significant predictor for heat pain threshold at baseline (P = 0.0148), while the IL-1β was positively associated with the change in the heat tolerance per 1-µg/ml-increase (analgesic potency) of remifentanil (P = 0.0256, Table 3).

**Table 3:** Linear regression results for the effect of sleep, inflammation, and hypoxia markers on heat pain at baseline and under remifentanil.

HEAT PAIN THRESHOLD	Baseline		Response to Remifentanil	
	Adjusted beta (SE)	P value	Adjusted beta (SE)	P value
**POLYSOMNOGRAM**				
WASO	−0.0023 (0.0117)	0.8423	0.0014 (0.0033)	0.6648
Stage shifts	−0.0066 (0.0101)	0.5134	−0.0006 (0.0030)	0.8316
Nadir SaO_2_	−0.0741 (0.0654)	0.2575	−0.0172 (0.0188)	0.3603
**INFLAMMATION MARKERS**				
IL-6	1.4336 (0.8897)	0.1071	−0.1124 (0.2693)	0.6763
IL-1β	0.7990 (0.6320)	0.2061	−0.0602 (0.1858)	0.7460
TNF-α	−0.0443 (0.1409)	0.7530	−0.0336 (0.0419)	0.4218
**HYPOXIA MARKERS**				
IGFBP-1	0.0003 (0.0001)	0.0148	−0.0001 (0.0000)	0.0524
EPO	−0.0280 (0.1217)	0.8177	0.0130 (0.0357)	0.7155
VEGF	−0.0016 (0.0015)	0.2732	0.0005 (0.0004)	0.2532
**HEAT PAIN TOLERANCE**				
**POLYSOMNOGRAM**				
WASO	0.0106 (0.0105)	0.3132	−0.0030 (0.0031)	0.3302
Stage shifts	−0.0056 (0.0091)	0.5357	0.0044 (0.0028)	0.1223
Nadir SaO_2_	0.0125 (0.0590)	0.8319	−0.0129 (0.0176)	0.4659
**INFLAMMATION MARKERS**				
IL-6	0.4913 (0.8015)	0.5399	−0.1904 (0.2511)	0.4484
IL-1β	0.2408 (0.5695)	0.6344	0.3899 (0.1747)	0.0256
TNF-α	−0.1046 (0.1269)	0.4100	−0.0493 (0.0395)	0.2116
**HYPOXIA MARKERS**				
IGFBP-1	0.0002 (0.0001)	0.1165	0.0000 (0.0000)	0.3738
EPO	0.1071 (0.1096)	0.3284	0.0405 (0.0336)	0.2282
VEGF	−0.0011 (0.0013)	0.3878	0.0000 (0.0004)	0.9962

Sleep continuity and breathing polysomnographic (PSG) descriptors like the wake after sleep onset (WASO) time, number of sleep stage shifts, and the lowest recorded SaO_2_ during sleep (nadir SaO_2_), as well as basic inflammation and hypoxia markers were employed as predictors of heat pain-related variables (threshold and tolerance) in the model. At baseline, betas (standard errors, SE) for the various predictors in the regression equation reflect the change in the heat pain threshold and tolerance for every one unit of change in the PSG and inflammatory markers. Under remifentanil, betas reflect the change in the analgesic sensitivity to remifentanil for the heat pain threshold and tolerance. For example, for every 1-pg/ml-increase in the serum IL-1β the heat pain tolerance will additionally increase by 0.3899°C for every 1-µg/ml-increase in the target Ce of remifentanil. Betas for the PSG predictors were adjusted for the inflammatory and hypoxia markers. Alpha level was set at 0.05.

Nadir SaO_2_ was negatively associated with the analgesic potency of remifentanil for the cold pain threshold (P = 0.0440). Serum TNF-α was positively associated with the analgesic potency of remifentanil for the cold pain threshold (P = 0.0276), while IL-1β was positively associated with the analgesic potency of remifentanil for cold pain tolerance (P = 0.0218). From the hypoxia markers, IGFBP-1 was positively associated with both the baseline cold pain threshold (P = 0.0013) and the analgesic potency of remifentanil (P = 0.0018, Table 4).

**Table 4:** Linear regression results for the effect of sleep, inflammatory, and hypoxia-related markers on cold-induced pain at baseline and under remifentanil.

COLD PAIN THRESHOLD	Baseline		Response to Remifentanil	
	Adjusted beta (SE)	P value	Adjusted beta (SE)	P value
**POLYSOMNOGRAM**				
WASO	0.0300 (0.0421)	0.4764	0.0815 (0.0838)	0.3307
Stage shifts	0.0174 (0.0347)	0.6161	−0.0564 (0.0777)	0.4680
Nadir SaO_2_	−0.1601 (0.2307)	0.4875	−0.9694 (0.4813)	0.0440
**INFLAMMATION MARKERS**				
IL-6	−1.5441 (3.0639)	0.6143	−4.6060 (6.8895)	0.5038
IL-1β	−3.3280 (2.2038)	0.1310	−0.3734 (4.7578)	0.9374
TNF-α	−0.1410 (0.4897)	0.7734	2.4017 (1.0801)	0.0262
**HYPOXIA MARKERS**				
IGFBP-1	0.0013 (0.0004)	0.0013	0.0025 (0.0008)	0.0018
EPO	0.7167 (0.4261)	0.0926	0.1360 (0.9198)	0.8824
VEGF	−0.0052 (0.0051)	0.3070	−0.0089 (0.0113)	0.4302
**COLD PAIN TOLERANCE**				
**POLYSOMNOGRAM**				
WASO	0.3645 (0.3801)	0.3376	−0.2252 (0.1543)	0.1443
Stage shifts	0.3535 (0.3236)	0.2746	−0.0820 (0.1430)	0.5663
Nadir SaO_2_	−2.9573 (2.1166)	0.1624	0.4166 (0.8831)	0.6371
**INFLAMMATION MARKERS**				
IL-6	−39.2327 (28.5353)	0.2042	0.2783 (12.6827)	0.9825
IL-1β	−21.6649 (20.3739)	0.2876	20.0845 (8.7405)	0.0216
TNF-α	2.1202 (4.5381)	0.6404	−2.0639 (1.9717)	0.2952
**HYPOXIA MARKERS**				
IGFBP-1	0.0034 (0.0037)	0.3571	0.0000 (0.0015)	0.9812
EPO	5.3931 (3.9169)	0.1685	−0.2520 (1.6766)	0.8805
VEGF	−0.0344 (0.0474)	0.4678	0.0034 (0.0207)	0.8711

Sleep continuity and breathing polysomnographic (PSG) descriptors like the wake after sleep onset (WASO) time, number of sleep stage shifts, and the lowest recorded SaO_2_ during sleep (nadir SaO_2_), as well as basic inflammation and hypoxia markers were employed as predictors of heat pain-related variables (threshold and tolerance) in the model. At baseline, betas (standard errors, SE) for the various predictors in the regression equation reflect the change in the cold pain threshold and tolerance for every one unit of change in the PSG and inflammatory markers. Under remifentanil, betas reflect the change in the analgesic sensitivity to remifentanil for the cold pain threshold and tolerance. For example, for every 1-%-absolute decrease in the nadir SaO_2_ the cold pain threshold will additionally increase by 0.9694°C for every 1-µg/ml-increase in the target Ce of remifentanil. Betas for the PSG predictors were adjusted for the inflammatory and hypoxia markers. Alpha level was set at 0.05.

## Discussion

We found that nocturnal hypoxemia, quantified by the lowest arterial oxyhemoglobin saturation (nadir SaO_2_) during sleep, was inversely proportional to the elevation of cold pain threshold under remifentanil infusion. Nocturnal hypoxemia thus potentiated the analgesic effect of an μ-opioid agonist. In corroboration of this finding, increased serum IGFBP-1, a hypoxia marker with an extensive research record in perinatal medicine [59]–[62], was predictive of decreased pain sensitivity and an enhanced potency for opioid analgesia. These findings confirm previous evidence in children suffering from obstructive sleep apnea (OSA), where the analgesic requirement for postoperative morphine was inversely proportional to the magnitude of nocturnal hypoxemia (lowest SaO_2_) experienced preoperatively [34], [35]. Furthermore, both children suffering from OSA [63] and rats treated during development with chronic intermittent hypoxia [64] were more sensitive to the respiratory depressant effect of fentanyl. Although the mechanism for this effect of recurrent hypoxia on pain and analgesia systems is vague, in vivo evidence suggests that an up-regulation of μ-opioid receptors might be responsible for the observed increase in the potency of opioids [65]–[67].

Nocturnal hypoxemia in OSA frequently results in, and is associated with disruption of sleep. Thus, polysomnographic (PSG) descriptors like apnea/hypopnea index, desaturation index, or arousal index may reflect physiological events that are associated with both sleep disruption and nocturnal hypoxemia. In our effort to dissect these two phenotypic components of OSA, we selected PSG indices like the number of sleep stage shifts and “wake after sleep onset” (WASO), which, although reflecting sleep disruption, were not correlated with arterial desaturation expressed by nadir SaO_2_. The use of nadir SaO_2_ as indicator of nocturnal hypoxemia was selected based on prior clinical evidence regarding its effect on the postoperative morphine requirement in children with OSA [34], [35].

Although sleep continuity was disturbed in subjects with a PSG-based diagnosis of OSA (*i.e.*, those with AHI≥5, Table 2), none of the examined parameters (Wake time After Sleep Onset and number of Sleep Stage Shifts) was significantly related to either pain behavior or opioid analgesic effect, even after adjusting our regression model for a home-based vs. in-laboratory PSG study. Previous evidence in healthy volunteers supports a hyperalgesic effect for sleep deprivation and sleep fragmentation [19]–[21], [23], [57], [68], [69]. Although this finding has been replicated in subjects suffering from primary insomnia [70], experimental testing demonstrated decreased sensitivity to pain in patients with OSA [58]. It may be that the two phenotypic components of OSA (i.e., sleep disruption and nocturnal hypoxemia) exert opposite effects on pain behavior; i.e., sleep disruption enhances, whereas nocturnal hypoxemia ameliorates pain. Therefore, depending on the severity of nocturnal hypoxemia, the effect of the latter could either mask the hyperalgesia produced by sleep disruption, or even result in hypoalgesia. Based on the presumed evidence that inadequate or fragmented sleep could promote systemic inflammation [21], [24], [30], [57], [71], another reason for the lack of an effect of sleep disruption on pain behavior and/or analgesia might have been the adjustment of the model for the presence of systemic inflammation, using IL-6, IL-1β, and TNF-α; however, the unadjusted model did not reveal any effect neither. Another potential confounder for the hyperalgesic effect of sleep disruption may be inquired in the evidence that physiological sleepiness (i.e., determined objectively by the Multiple Sleep Latency Test, MSLT) in otherwise healthy pain-free volunteers has been associated with an enhanced sensitivity to experimental heat pain [72] and decreased opioid potency for analgesia [73]. In the present experiment, we employed the Epworth sleepiness scale (ESS), a subjective instrument with a nonetheless excellent correlation with MSLT [74], to evaluate habitual sleepiness in our subjects. Median ESS scores were close to a generally accepted normal level (ESS = 6) and did not differ between subjects with and without OSA. In spite of the fact that certain subjects from both groups presented with excessive sleepiness scores (ESS>10), a further adjustment of our model for ESS (results not shown) did not change our basic findings. Finally, the relatively small number of subjects suffering from moderate (15≤ AHI<30, N = 7) and severe (AHI≥30, N = 6) OSA might have been responsible for our inability to detect associations between sleep disruption and pain behavior. Lastly, we need to recognize the fact that most of the study participants, possibly due to a *first night effect* [75], experienced a decreased amount of deep (stage 3 and 4) sleep, which might have also hindered our ability to detect an association between OSA-related inadequate/fragmented sleep and pain behavior.

Based on prior evidence for their involvement in the regulation of sleep function [25], [26] and in pro-inflammatory physiology of sleep-disordered breathing [18], [76], [77], we selected TNF-α, IL-1β, and IL-6, to include as the basic explanatory inflammatory markers in our model. We found that increased IL-1β in the serum significantly predicted enhanced sensitivity to heat pain (i.e., decreased pain threshold), while both IL-1β and TNF-α were positively associated with an augmented potency of remifentanil analgesia for heat and cold-induced pain. These findings are in agreement with molecular and clinical evidence that inflammation-related changes may result in hyperalgesia and an increased sensitivity to the analgesic effect of opioids [78]–[82].

Both TNF-α and IL-1β are up-regulated in the brain during prolonged wakefulness [83], while injection of exogenous TNF-α or IL-1β imitates, to a certain extent, the physiology of sleep loss by increasing propensity to sleep [25], [26], [84]. Both chronic intermittent hypoxia and sleep fragmentation selectively and independently activate major inflammatory pathways with increased TNF-α, and IL-6 in the serum of OSA patients [74], [77], [85]. Expression of the same inflammatory paths in volunteers, as result of total or partial sleep deprivation [24], [71] was associated with the development of spontaneous pain [21], [57], while TNF-α, IL-1β, and IL-6 have been implicated in the development of neuropathic pain through modification of peripheral receptor channels and/or induction of changes in the synaptic function of CNS neurons [31], [32], [82], [86]–[88]. Furthermore, molecular and in vivo evidence suggest that changes occurring in the milieu of afferent sensory neurons during inflammation, may augment the potency of μ-agonists either by up-regulation of μ-opioid receptors or by increasing the affinity of the latter for their exogenous or endogenous ligands [78], [81], [89].

Heat and cold pain-related measurements that were censored at 52°C and 180 sec, respectively, were included in the analysis without any special modeling. This statistical treatment consists a conservative approach, thus should preserve the validity of the reported findings. Considering the dimension of covariates in the fully adjusted regression model, the sample size of the current study is limited and the analysis results might be sensitive to specific model assumptions. We recognize the preliminary character of our investigation, which is potentially underpowered to detect some associations of interest. A larger study is needed to verify our findings as well as identify smaller yet still clinically meaningful and/or scientifically interesting associations.

In conclusion, we found that nocturnal hypoxemia in volunteers at high risk for obstructive sleep apnea was associated with an increased potency for opioid analgesia. A hypoxia marker like IGFBP-1 was associated with a decreased sensitivity to pain and increased potency to opioid analgesia, whereas pro-inflammatory markers also predicted an enhanced sensitivity to opioid analgesia.
